# Investigation of Molecular Mechanisms Involved in Sensitivity to the Anti-Cancer Activity of Costunolide in Breast Cancer Cells

**DOI:** 10.3390/ijms24044009

**Published:** 2023-02-16

**Authors:** Yu-Jeong Choi, Youn Kyung Choi, Seong-Gyu Ko, Chunhoo Cheon, Tai Young Kim

**Affiliations:** 1 Department of Science in Korean Medicine, Graduate School, Kyung Hee University, Seoul 02447, Republic of Korea; 2 Jeju Research Center for Natural Medicine, Jeju National University, Jeju 63243, Republic of Korea; 3 Department of Preventive Medicine, College of Korean Medicine, Kyung Hee University, Seoul 02447, Republic of Korea; 4 Center for Cognition and Sociality, Institute for Basic Science, Daejeon 34126, Republic of Korea

**Keywords:** costunolide, breast cancer, ROS, lysosomal membrane permeabilization (LMP), mitochondrial apoptosis, mitophagy

## Abstract

Costunolide (CTL), an active compound isolated from *Saussurea lappa* Clarke and *Laurus nobilis* L, has been shown to induce apoptosis via reactive oxygen species (ROS) generation in various types of cancer cells. However, details of molecular mechanisms underlying the difference in sensitivity of cancer cells to CTL are still largely unknown. Here, we tested the effect of CTL on the viability of breast cancer cells and found that CTL had a more efficient cytotoxic effect against SK-BR-3 cells than MCF-7 cells. Mechanically, ROS levels were significantly increased upon CTL treatment only in SK-BR-3 cells, which leads to lysosomal membrane permeabilization (LMP) and cathepsin D release, and subsequent activation of the mitochondrial-dependent intrinsic apoptotic pathway by inducing mitochondrial outer membrane permeabilization (MOMP). In contrast, treatment of MCF-7 cells with CTL activated PINK1/Parkin-dependent mitophagy to remove damaged mitochondria, which prevented the elevation of ROS levels, thereby contributing to their reduced sensitivity to CTL. These results suggest that CTL is a potent anti-cancer agent, and its combination with the inhibition of mitophagy could be an effective method for treating breast cancer cells that are less sensitive to CTL.

## 1. Introduction

Breast cancer is the most common cancer in women, and its incidence rates are still increasing in the United States and many countries in Asia [1,2]. Targeted therapy using monoclonal antibodies has proven effective in treating breast cancer; however, chemotherapy remains an important option in breast cancer treatment along with surgery and radiation therapy [3,4,5]. Reactive oxygen species (ROS) are constantly generated as by-products during mitochondrial aerobic respiration and can be beneficial or harmful for cell growth, depending on their intracellular levels [6,7]. Cancer cells maintain ROS within a certain level by using several antioxidant systems and utilize them as a second messenger for activating redox-sensitive signaling pathways to promote their proliferation [8]. However, enhancement of intracellular ROS levels above a tolerable limit can kill cancer cells, and thus pharmacological agents that increase ROS have been suggested as potential anti-cancer drugs. In fact, high levels of ROS generated by chemotherapy regimens of various drugs, including doxorubicin and cisplatin, are known to contribute to the death of breast cancer cells [9,10,11].

One of the common mechanisms by which ROS promotes cancer cell death is the activation of mitochondria-dependent apoptotic pathways involving Bcl-2 family proteins [12,13]. Under normal conditions, anti-apoptotic B-cell lymphoma-2 (Bcl-2) protein binds and inactivates pro-apoptotic Bcl-2-associated X protein (Bax) and Bcl-2 antagonist/killer (Bak), whereas under oxidative stress conditions, Bcl-2 is downregulated and Bax is translocated from the cytosol to the mitochondria and homo- or hetero-oligomerize with Bax or Bak to form pores, which lead to mitochondrial outer membrane permeabilization (MOMP), causing a release of pro-apoptotic cytochrome c into the cytosol and subsequent activation of the caspase cascade [14]. The formation of BAX homo-oligomers can also be triggered by tBID, a truncated form of Bid [15], and the cleavage of Bid requires caspase-8, -10, or lysosomal proteases, such as cathepsin D [16,17].

Costunolide (CTL), a sesquiterpene lactone, is a natural product derived from the plants *Saussurea lappa* Clarke and *Laurus nobilis* L. (Lauraceae) [18]. Previous studies have shown that CTL can efficiently induce programmed cell death, such as apoptosis and autophagy, mainly through ROS- and AKT-mediated signaling pathways in various cancers [19,20,21,22]. Similarly, CTL exerts its anti-cancer activity on breast cancer cells by targeting several signaling cascades associated with cell cycle arrest and apoptosis [23,24,25]. However, studies reporting the effect of CTL on ROS generation in breast cancer cells are limited, and investigating molecular pathways that determine the sensitivity of cancer cells to CTL is lacking. In this study, we observed that breast cancer cells displayed differential sensitivity to CTL depending on the level of ROS production. Thus, we aimed to investigate the signaling pathways that mediate susceptibility to CTL in these breast cancer cells.

## 2. Results

### 2.1. CTL Promotes Apoptotic Breast Cancer Cell Death in a ROS-Dependent Manner

**Figure 1:**
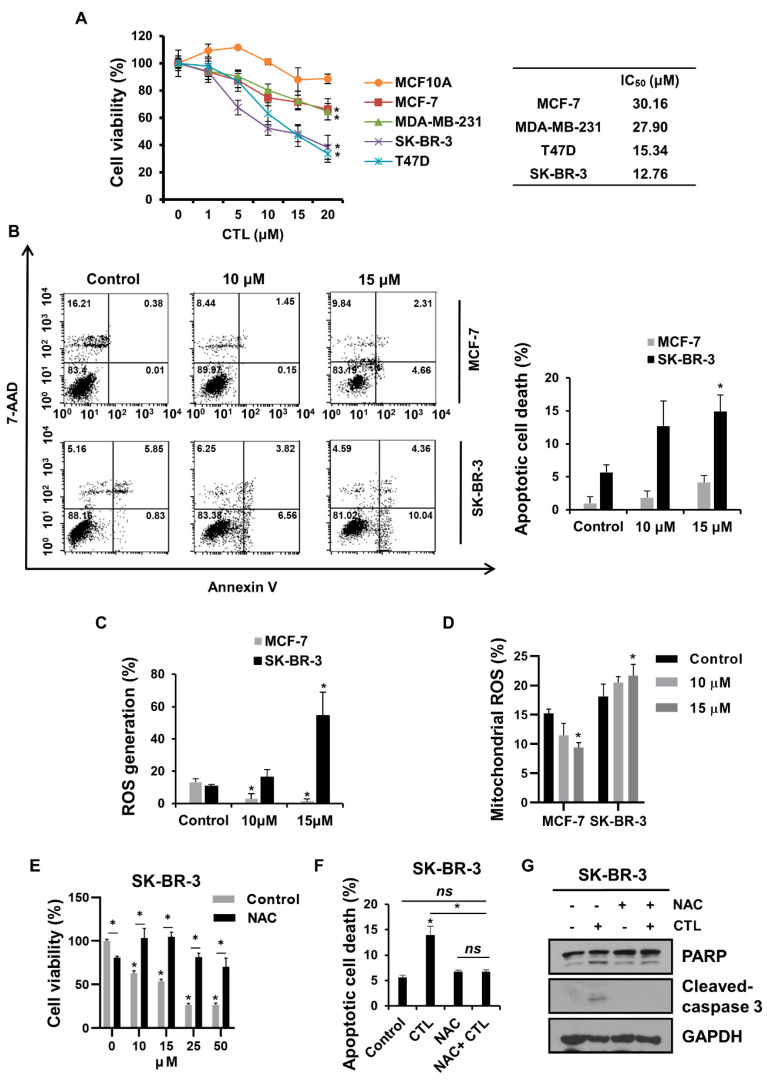
CTL promotes ROS-dependent apoptosis in breast cancer cells. (**A**) Breast cancer cells were treated with indicated concentrations of CTL for 48 h. The cell viability was measured by the MTT assay. Statistical significance compared to the control group is indicated by * (*p* < 0.05). (**B**) Cells were stained with Annexin V//7-AAD dye and analyzed by flow cytometry using CellQuest Pro software version 5.2. The bar plot was quantified by three independent experiments. (**C**) Cells were collected and stained with CTL for 1 h and then incubated with DCFH-DA for 30 min. ROS levels were measured by flow cytometry. (**D**) Mitochondrial ROS was detected using the MitoSOX reagent. Cells were treated with CTL for 1 h and stained with MitoSOX dye for 30 min. MitoSOX fluorescence was measured by flow cytometry. (**E**) After treatment with 5mM NAC for 1 h, cells were treated with CTL for 48 h. Cell viability was analyzed using the MTT assay. (**F**,**G**) SK-BR-3 cells were pre-treated with 5 mM NAC for 1 h, followed by being treated with or without 15 μM CTL for 24 h. Apoptosis was analyzed by flow cytometry. The level of PARP and cleaved caspase 3 was detected by western blotting. Data represent the mean ± SD. *, *p* < 0.05 using the Student’s *t*-test or one-way ANOVA with Bonferroni post hoc test. ns—not significant.

We first investigated the effects of CTL on cell growth in four breast cancer cells, such as SK-BR-3, T47D, MCF-7, and MDA-MB-231, as well as MCF10A, normal mammary epithelial cells. MTT assay showed that CTL suppressed the growth of all breast cancer cells, but the inhibitory effect was more prominent in SK-BR-3 and T47D than MCF-7 and MDA-MB-231 with IC_50_ values of about 12.76 μM and 15.34 μM versus 30.16 μM and 27.90 μM, respectively (Figure 1A). Importantly, CTL did not exhibit significant cytotoxic activity against MCF-10A even at the concentration of 20 μM, indicating a potential of CTL as a cancer-specific therapeutic agent. Here, SK-BR-3 and MCF-7 cells were chosen for further experiments. Next, we investigated the effects of CTL on apoptotic cell death of these cells, and it was observed that CTL was more effective in inducing apoptosis in SK-BR-3 cells than in MCF-7 cells (Figure 1B). These results indicate that these breast cancer cells have different sensitivities to CTL. Previous studies demonstrated that CTL induces apoptotic cancer cell death in renal cell carcinoma and osteosarcoma cancers through ROS production [20,26]. Therefore, we sought to examine whether CTL also promotes intracellular ROS generation in breast cancer cells by staining with DCFH-DA, a ROS indicator. Similar to previous results, our data also showed that CTL significantly increased the level of intracellular ROS in SK-BR-3 cells (Figure 1C). Moreover, we evaluated the effects of CTL on mitochondrial ROS generation using MitoSOX, a mitochondrial superoxide indicator, and found that the MitoSOX fluorescent signal was increased upon CTL treatment only in the high-sensitive breast cancer cells (Figure 1D and Figure S1). Meanwhile, both intracellular and mitochondrial ROS were rather decreased after CTL treatment in the low-sensitive breast cancer cells (Figure 1C,D and Figure S1). It is important to note that MCF-7 and T47D cells displayed different sensitivity to CTL and mitochondrial ROS production even though both cells are ER+ luminal subtypes, implicating that drug sensitivity of breast cancer cells to CTL is independent of their subtype but dependent on ROS production. These results suggest that ROS level might be a key factor in determining CTL sensitivity in breast cancer cells. To test this possibility, we pre-treated NAC, a ROS inhibitor, prior to CTL treatment. Importantly, NAC partially rescued cell viability but almost completely rescued apoptotic cell death induced by CTL in SK-BR-3 cells (Figure 1E,F). The strong rescuing effect of NAC on CTL-induced apoptosis was confirmed by western blot analysis that the increased levels of apoptotic markers such as cleaved-PARP and -caspase 3 by CTL treatment were blocked by NAC pre-treatment in SK-BR-3 (Figure 1G). Taken together, our results demonstrate that CTL induces apoptotic cell death to a different degree in breast cancer cells, and the sensitivity is correlated with CTL-induced ROS production.

### 2.2. CTL-Mediated ROS Production Induces LMP and the Release of Cathepsin D in SK-BR-3 Cells

**Figure 2:**
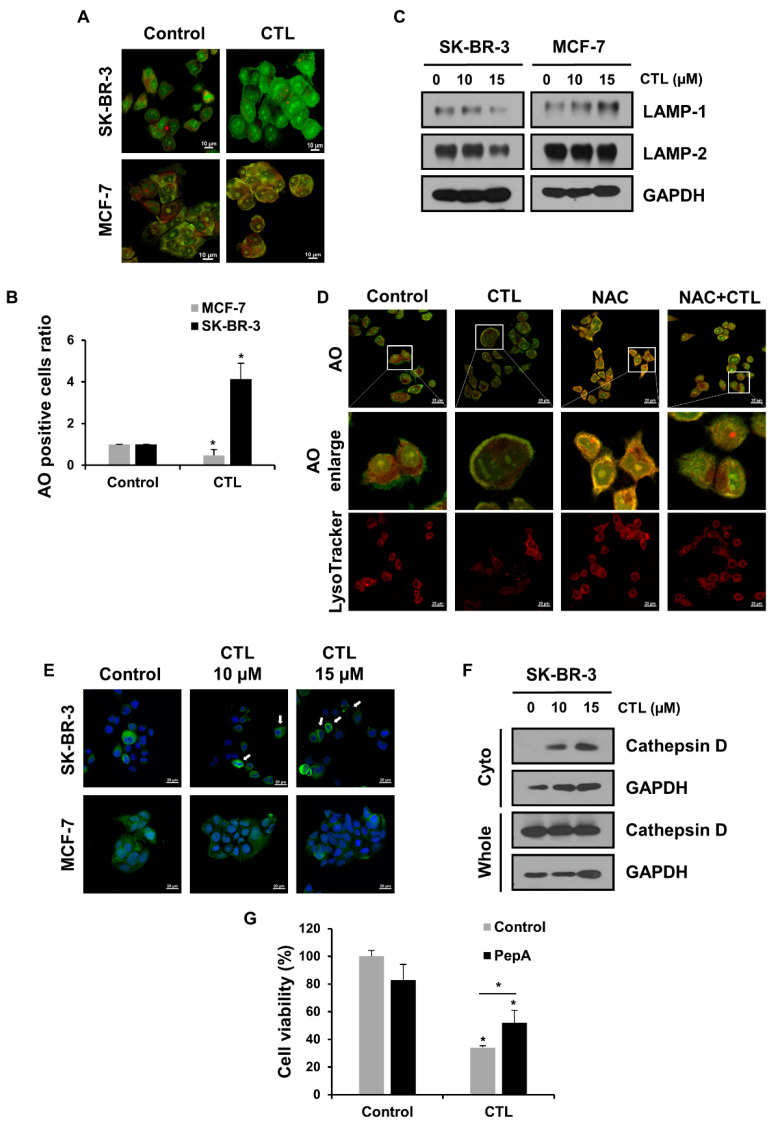
ROS produced by CTL induces LMP, and the lysosomal protease cathepsin D leads to cell death in SK-BR-3 cells. (**A**,**B**) Cells were treated with CTL (15 μM) for 24 h and then incubated with 2 μg/mL acridine orange (AO) for 10 min. For immunofluorescence detection, stained cells were fixed with 4% PFA, and lysosome stability was observed using a confocal microscope. For flow cytometry analysis, cells were stained with AO dye and harvested, and fluorescence intensities were measured in the green channels. Statistical significance was calculated using Student’s *t*-tests. *, *p* < 0.05. (**C**) Lysosomal membrane proteins were detected in the cells exposed to CTL for 24 h. (**D**) SK-BR-3 cells were treated with 15 μM CTL for 24 h in the presence or absence of NAC. Lysosome stability was assessed by staining with AO and Lysotracker Red DND-99. Cells were treated with 2 μg/mL AO for 10 min and 50 nM Lysotracker Red DND-99 for 30 min, respectively. Images were obtained by confocal microscopy. The image of the white rectangles highlights enlarged image. (**E**) Cells were stained with anti-cathepsin D (green) and DAPI (blue) after exposure to CTL for 24 h. The release of cathepsin D into the cytosol was detected by a confocal microscope. Arrows indicate cathepsin D released into the cytosol. (**F**) SK-BR-3 cells were treated with CTL for 24 h. Cellular components were separated with cytosolic (Cyto) fraction, and the increased cathepsin D in the cytosol was analyzed by western blotting. (**G**) SK-BR-3 cells were treated with PepA (50 μM) for 1 h and then incubated with CTL at 15 μM for 48 h. Cell viability was determined by MTT assay. Data represent the mean ± SD, and statistical analysis was performed using one-way ANOVA followed by a Bonferroni post-hoc test. *, *p* < 0.05.

Previous studies reported that high levels of ROS cause lysosomal membrane permeabilization (LMP), whereby lysosomal cathepsins are released to the cytosol and promote cell death [27,28]. We first investigated whether CTL can induce LMP in breast cancer cells using acridine orange (AO). This fluorescent dye is accumulated in acidic organelles and emits red fluorescence but undergoes a shift to green fluorescence upon its release into the cytosol during LMP induction [29]. Confocal imaging of AO staining showed that after treatment of CTL, green fluorescence was evidently observed in SK-BR-3 cells, but it was rarely detected in MCF-7 cells (Figure 2A). Flow cytometry confirmed that the number of green fluorescence-positive cells was increased upon CTL treatment in SK-BR-3 cells, not in MCF-7 cells, indicating that CTL induces the lysosome disruption in only CTL-sensitive SK-BR-3 cells (Figure 2B). Previous studies have shown that lysosomal cell death can be enhanced by inhibiting the lysosome-associated membrane proteins (LAMPs) [30,31]. Consistent with these results, CTL suppressed the expression of LAMP-1 and -2 in SK-BR-3 cells but did not affect or increase their level in MCF-7 cells (Figure 2C). We further assessed whether the CTL-induced rupture of the lysosomal membrane in SK-BR-3 cells is due to the ROS production seen in Figure 1C,D. Importantly, pre-treatment of SK-BR-3 cells with NAC abolished the CTL-induced AO fluorescence switching from red to green (Figure 2D). Similar results were obtained when LysoTracker red was used to visualize acidic compartments (Figure 2D). We next asked whether the CTL-induced LMP leads to the leakage of lysosomal proteases by immunostaining. As shown in Figure 2E, the cathepsin D was translocated to the cytosol upon CTL treatment, which was clearly seen only in SK-BR-3 cells. Cell fractionation studies confirmed the release of cathepsin D into the cytosol after CTL treatment in these cells (Figure 2F). Finally, we determined whether the released cathepsin D contributes to SK-BR-3 cell death using the lysosomal protease inhibitor, pepstatin A (PepA). The extent of cell death seen in the presence of CTL was significantly reversed by co-treatment with PepA (Figure 2G). Overall, these results suggest that ROS production by CTL leads to the LMP-mediated cathepsin D release, which results in SK-BR-3 cell death.

### 2.3. The Released Cathepsin D Leads to MOMP and Subsequent Mitochondrial Apoptosis in SK-BR-3 Cells

**Figure 3:**
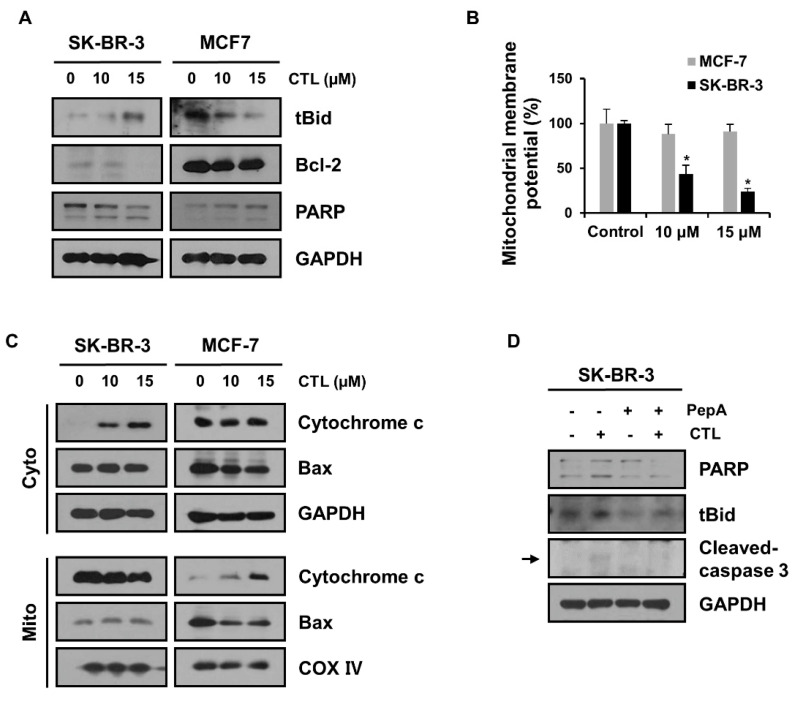
CTL-induced mitochondria apoptosis is dependent on the cathepsin D in SK-BR-3 cells. (**A**) Cells were treated with CTL for 24 h, and protein expressions were detected via western blotting. (**B**) The mitochondrial membrane potential was measured by rhodamine 123 staining (1 μg/mL). The fluorescence intensity was measured by flow cytometry. (**C**) After 24 h treatment with CTL, cells were separated into cytosolic (Cyto) and mitochondria (Mito) fractions. Cytochrome c and Bax release into the cytoplasm or mitochondria fraction were analyzed by western blotting. (**D**) SK-BR-3 cells were treated with PepA (50 μM) for 1 h and then incubated with CTL (15 μM). Protein levels of pro-apoptosis markers were analyzed using western blotting. The arrow indicates the band corresponding to cleaved caspase 3. Data represent the mean ± SD. *, *p* < 0.05 using Student’s *t*-tests.

It has been reported that cathepsin D released from the lysosome promotes the degradation of anti-apoptotic Bcl-2 protein and the cleavage of Bid to tBid, which recruits BAK to the mitochondrial outer membrane and induces its oligomerization. The Bax oligomers, in turn, engage MOMP and cytochrome c release, leading to mitochondrial apoptosis [32,33,34]. To investigate the effects of CTL on these pathways, we performed a western blot analysis and observed that CTL treatment decreased Bcl-2 expression and increased tBid levels in SK-BR-3 cells (Figure 3A). We further found that CTL markedly reduced mitochondrial membrane potential (MMP) in SK-BR-3 cells, as measured by flow cytometry analysis using mitochondria probe, Rhodamine 123 (Figure 3B), which might be a result of the induction of MOMP [35,36]. Note that these mitochondrial apoptotic pathways were not triggered in the CTL-treated MCF-7 cells (Figure 3A,B). Next, to examine the effect of CTL on the Bax-dependent cytochrome c release, we performed subcellular fractionations and western blot analysis of mitochondrial and cytoplasmic fractions. Upon treatment with CTL, mitochondrial Bax translocation and cytosolic release of cytochrome c were enhanced in SK-BR-3 cells. However, these pro-apoptotic effects were not observed in MCF-7 cells (Figure 3C). Finally, to demonstrate that the released cathepsin D is the main mediator of mitochondrial apoptosis in the CTL-treated SK-BR-3 cells, we used PepA. Co-treatment with PepA abrogated the CTL-induced increase in the cleavage of tBid, PARP, and caspase 3 (Figure 3D). These data suggest that cathepsin D released from the lysosome by CTL promotes mitochondrial apoptosis via induction of MOMP and cytochrome C release in the CTL-sensitive SK-BR-3 cells.

### 2.4. Mitophagy Protects MCF-7 Cells from CTL-Induced Mitochondrial Damage and Cell Death

**Figure 4:**
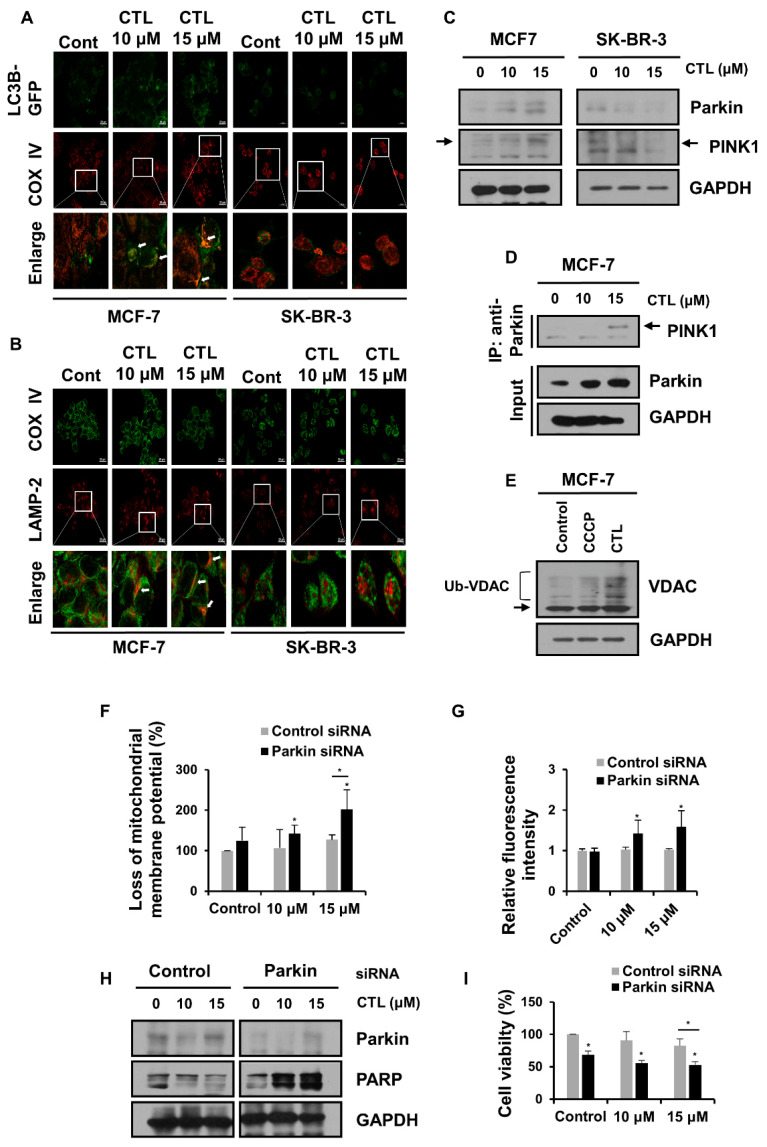
MCF-7 cells activate PINK1/Parkin-dependent mitophagy as a defense mechanism against CTL-induced ROS production. (**A**) MCF-7 and SK-BR-3 cells were transfected with LC3-GFP (autophagosome marker, green) for 24 h and treated with CTL for another 24 h. Cells were immunostained with anti-COX IV (mitochondrial marker, red). (**B**) Cells were treated with CTL for 24 h and then stained with anti-COX IV (green) and anti-LAMP2 (lysosome marker, red). The images were analyzed using confocal microscopy (Carl Zeiss, Jena, Germany) at ×40 magnification. The white rectangle highlights the magnified merged image. Arrows indicate the colocalization part. (**C**) After CTL treatment for 24 h, mitophagy proteins were detected by western blotting. (**D**) MCF-7 cell lysates exposed to CTL for 24 h were immunoprecipitated using anti-Parkin and analyzed by immunoblotting with the indicated antibodies. (**E**) MCF-7 cells were treated with CCCP (50 µM) and CTL (15 µM) for 24 h. Immunoblot was shown the mitochondrial protein VDAC and ubiquitylated VDAC (Ub-VDAC). (**F**–**I**) MCF-7 cells were transfected with siRNA against Parkin and then treated with CTL for 24 h. (**F**) Cells were stained with rhodamine 123 (1 μg/mL) for 1 h, and loss of MMP was analyzed by flow cytometry. (**G**) The measurement of DCFH-DA fluorescence to detect ROS production was analyzed using a microplate reader (Biotek, Korea). (**H**) Protein levels were analyzed by western blotting. (**I**) Cell viability analysis was performed by MTT assay. Statistical analyses were performed Student’s *t*-tests or one-way ANOVA followed by Bonferroni post-hoc test. *, *p* < 0.05.

Given that MCF-7 cells did not produce ROS but rather decreased its level upon CTL treatment (Figure 1C), we speculated that MCF-7 cells might actively operate a defense mechanism against ROS production, which could be the reason for the observed lower sensitivity of MCF-7 cells to CTL compared to SK-BR-3 cells. Mitophagy is a selective form of autophagy that removes damaged mitochondria, a major source of aberrant ROS production [37]. We thus tested whether mitophagy is induced after CTL treatment in MCF-7 cells. Confocal microscopy showed that the number of autophagosomes labeled with GFP-LC3B was increased and colocalized with cytochrome c oxidase IV (COX IV), a mitochondria protein in MCF-7 cells, upon CTL treatment (Figure 4A). In addition, an increase in colocalization of COX IV with LAMP-2 was observed in the CTL-treated MCF-7 cells (Figure 4B). On the other hand, SK-BR-3 cells did not show the overlap between these mitochondria protein and autophagy components upon CTL treatment (Figure 4A,B). Next, we examined the effect of CTL on the PINK1-Parkin pathway of mitophagy which is known to be activated in response to oxidative stress [38,39]. When the mitochondria are damaged, PINK1 is stabilized on the outer membrane of mitochondria and recruits Parkin, which ubiquitinates a number of outer mitochondrial membrane proteins and brings autophagic vesicles to the ubiquitinated mitochondrial proteins [40]. We found that PINK1 and Parkin were upregulated by CTL treatment in MCF-7 cells but downregulated in SK-BR-3 cells (Figure 4C). Moreover, the coimmunoprecipitation assay revealed that the interaction between PINK1 and Parkin was increased after CTL treatment in MCF-7 cells (Figure 4D). We further found that CTL leads to ubiquitination of voltage-dependent anion channel (VDAC), mitochondrial outer membrane protein, in these cells (Figure 4E). The mitochondrial membrane uncoupler, carbonyl cyanide m-chlorophenylhydrazone (CCCP), was used as a positive control that induces PINK1/Parkin-mediated mitophagy [41,42]. Next, to investigate the role of PINK1-Parkin-mediated mitophagy in the reduced sensitivity to CTL in MCF-7 cells, a siRNA-mediated knockdown of Parkin was conducted. As shown in Figure 4F, CTL increased the loss of MMP in Parkin-depleted MCF-7 cells compared to control siRNA-transfected MCF-7 cells. More significantly, ROS levels were increased by CTL treatment in the Parkin-depleted MCF-7 cells (Figure 4G). Additionally, the knockdown of Parkin markedly elevated PARP cleavage and simultaneously decreased cell viability upon CTL treatment in MCF-7 cells (Figure 4H,I). Taken together, these findings indicate that mitophagy is a protective mechanism against ROS that confers reduced sensitivity to CTL in MCF-7 cells.

## 3. Discussion

Cancer cells produce more ROS than normal cells due to their elevated energy expenditure, resulting in a higher basal level of ROS [43]. This characteristic feature of cancer cells gives them a narrow window to counteract increased oxidative stress and, thus, selectively more vulnerable to ROS-producing agents. The mechanisms by which ROS induces cancer cell death are diverse, including apoptosis, cell cycle arrest, and autophagy [12]. CTL, an active compound derived from *Saussurea lappa* Clarke and/or *Laurus nobilis* L, has been reported to induce ROS generation in various cancer cells, such as breast, prostate, gastric, oral, and colon cancer cells, which leads to anti-cancer effects by promoting apoptosis or autophagy [21,44,45,46,47,48]. Our previous studies also demonstrated that CTL inhibits breast cancer cell growth and metastasis by causing ROS-induced cell cycle arrest at the G2/M phase and inhibiting TNFα-induced NF-κB activation in both in vitro and in vivo models [44,49]. Despite these previous studies, our understanding of how ROS produced by CTL induces cell death in breast cancer cells is limited. More importantly, the detailed molecular mechanisms regulating cancer cell sensitivity to CTL are lacking. Here, we found that two breast cancer cell lines, SK-BR-3 and MCF-7, exhibited different levels of sensitivity to CTL and thus focused on investigating the involved molecular pathways.

In this study, we observed that SK-BR-3 cells were more sensitive to CTL-induced apoptosis than MCF-7 cells. Intriguingly, ROS production was highly increased after CTL treatment only in the CTL-sensitive SK-BR-3 cells. Co-treatment with NAC, a ROS scavenger, abrogated the pro-apoptotic events in these cells, suggesting that ROS is a determining factor for the sensitivity of breast cancer cells to CTL. Excessive ROS can damage membrane structure and function by membrane lipid peroxidation. One of the major targets is the mitochondria membrane, whereby ROS triggers the opening of mitochondria permeability transition pore (MPTP) and disaggregates complexes I and III of the respiratory chain, leading to MOMP and cytochrome c release from mitochondria, which ultimately results in apoptosis [50,51]. ROS can also damage the lysosomal membrane, which leads to cathepsin release, promoting proteolytic activation of Bid and subsequent Bax-mediated cytochrome c release from mitochondria [30,32]. Thus, lysosomal membrane destabilization has been suggested to function upstream of MOMP, amplifying a critical event in the mitochondria-dependent apoptosis pathways [52,53]. This lysosome–mitochondria crosstalk was found in SK-BR-3 cells upon CTL treatment as demonstrated by CTL-induced lysosomal membrane rupture caused leakage of lysosomal cathepsin D, consequently activating mitochondrial apoptosis pathway through Bid cleavage.

One of the important findings of this study is that, in MCF-7 cells that are less sensitive to CTL, ROS was not increased but rather decreased after CTL treatment, implicating that active defense mechanisms for eliminating excessive ROS were turned on in these cells. We further found that mitophagy, an autophagy process that selectively removes damaged mitochondria as the main source of aberrant ROS production, was activated upon CTL treatment, suggesting that mitophagy contributes to the reduced sensitivity to CTL in MCF-7 cells. This idea is supported by our data demonstrating that the knockdown of Parkin, a key molecule in the mitophagy pathway, increased ROS levels and decreased cell viability upon CTL treatment in these cells. It is important to note that CTL damages the mitochondrial membrane but not the lysosomal membrane in MCF-7 cells, indicating that the lysosomal damages observed in CTL-treated SK-BR-3 might be caused by ROS originating from the damaged mitochondria due to CTL treatment. Therefore, we propose the model that CTL initially targets mitochondria, and then ROS generated later from the dysfunctional mitochondria damages lysosomal membranes, which further amplifies mitochondrial apoptosis signaling events in SK-BR-3 cells. On the other hand, MCF-7 cells actively remove damaged mitochondria through mitophagy to limit the spread of mitochondrial ROS to other cellular compartments. To our knowledge, our findings, for the first time, show the involvement of lysosomes in the CTL-mediated apoptosis pathway and a role for mitophagy in the reduced sensitivity of breast cancer cells to CTL. Whether these cellular responses to CTL exist only in breast cancer cells or are common in other cancers as well requires further investigation. Importantly, consistent with our observations, recent studies have reported that mitophagy contributes to the acquisition of resistance against various anti-cancer drugs, including cisplatin and sorafenib, and demonstrated that pharmacological inhibition or genetic ablation of mitophagy components such as PINK1, BNIP3, and ATAD3 sensitizes cancer cells to chemotherapy-induced cell death [54,55,56,57]. Therefore, we suggest mitophagy inhibition as a potential strategy to sensitize breast cancer cells to CTL.

Notably, these findings do not exclude the involvement of other cellular defense mechanisms against ROS in the CTL-low-sensitive breast cancer cells. We found that CTL increased the level of Nrf2, a key nuclear transcription factor that induces antioxidant enzymes [58], in the low-sensitive but not in the high-sensitive breast cancer cells (Figure S2), indicating that the Nrf2-mediated antioxidant response can be another mechanism employed by the CTL low-sensitive cells that contribute to scavenging excessive ROS. The precise mechanism requires further investigation in the future.

## 4. Materials and Methods

### 4.1. Chemicals

Costunolide (CTL) was purchased from Santa Cruz Biotechnology (Santa Cruz, CA, USA) and dissolved at a concentration of 100 mM in dimethyl sulfoxide (DMSO) (Sigma, St. Louis, MO, USA). The 3-(4,5-dimethylthiazol-2-yl)-2,5-diphenyltetrazolium bromide (MTT), N-Acetyl-L-cysteine (NAC), 2′,7′-Dichlorofluorescin diacetate (DCFH-DA), Carbonyl cyanide 3-chlorophenylhydrazone (CCCP), and Rhodamine 123 (Rho 123) were purchased from Sigma (St. Louis, MO, USA). Pepstatin A was obtained from Santa Cruz Biotechnology (Santa Cruz, CA, USA). Acridine Orange (AO), LysoTracker Red DND-99, and MitoSOX were purchased from Invitrogen (Carlsbad, CA, USA).

### 4.2. Cell Culture and Cell Viability Assay

Human MCF-7 and SK-BR-3 cells were obtained from Korean Cell Line Bank (Seoul, Republic of Korea). Cells were maintained in RPMI-1604 media (WelGENE Inc., Daegu, Korea) supplemented with 10% fetal bovine serum (FBS, J R Scientific, Woodland, CA, USA) and 1× penicillin-streptomycin solution (WelGENE Inc., Daegu, Republic of Korea) at 37 °C/5% CO_2_. Cell viability was examined using an MTT assay. Briefly, cells were seeded in 96-well at 5 × 10^3^ cells per well. Drugs were treated with indicated doses. Each well had 20 µL MTT solution (5 mg/mL) added for 2 h, then 100 µL DMSO was added at 37 °C for 2 h. The absorbance was determined at 570 nM using an ELISA plate reader (Molecular Devices, San Jose, CA, USA).

### 4.3. Western Blot Analysis

Collected cells were lysed into 2× sample buffer (ELPIS Biotech Inc., Daejeon, Republic of Korea) and denatured at 100 °C for 10 min. Protein extracts were separated on SDS-PAGE and transferred to a nitrocellulose membrane (GE Healthcare Life Sciences, Marlborough, MA, USA). The membranes were blocked with 5% skim milk in PBS with 0.1% Tween-20 (PBS-T) for 1 h, proved with primary antibodies and followed by incubation with secondary anti-rabbit IgG or -mouse IgG (SeraCare Life Sciences, Milford, MA, USA). The bands were detected using EZ-Western Lumi Pico Kit (DoGen, Seoul, Republic of Korea). The following primary antibodies were used: Bax (sc-7480, Santa Cruz, CA, USA), Bcl-2 (sc-7382, Santa Cruz, CA, USA), tBid (ab10640, Abcam, Cambridge, MA, USA), PARP (#9542, Cell Signaling Technology, Danvers, MA, USA), Cytochrome C (BD556433, BD Biosciences, San Jose, CA, USA), Cox IV (#4850, Cell Signaling Technology, Danvers, MA, USA), LAMP-1 (sc-20011, Santa Cruz, CA, USA), LAMP-2 (sc-18822, Santa Cruz, CA, USA), PINK1 (BC100-494, Novus Biologicals, Centennial, CO, USA), Parkin (#4211, Cell Signaling Technology, Danvers, MA, USA), VDAC (#4866, Cell Signaling Technology, Danvers, MA, USA), Ub (07-375, Sigma, St. Louis, MO, USA), and GAPDH (#5174, Cell Signaling Technology, Danvers, MA, USA).

### 4.4. Cell Fractionation

The proteins of cytosolic and mitochondrial fraction were prepared using a Cell Fraction Kit (Abcam, MA, USA) according to the manufacturer’s instructions. 2.5 × 10^6^ cells were treated with CTL for 24 h, harvested, and washed with 1× Buffer A. The supernatant was centrifuged at 10,000× *g* at 4 °C for 1 min, and the cytosolic and mitochondria fractions were separated in turn. Each protein concentration was measured by the Bradford assay (Bio-Rad, Hercules, CA, USA). A Cox IV or GAPDH was used as a loading control of mitochondria and cytosol, respectively.

### 4.5. Apoptosis Analysis

Cells were treated with CTL for 24 h, harvested, and washed in PBS. Cells resuspended with binding buffer were stained with Annexin V/7-AAD for 15 min at room temperature. After staining, cells were detected by FACSCalibur (BD Biosciences, San Jose, CA, USA). Apoptotic cells were analyzed using CellQuest Pro software version 5.2 (BD Biosciences, San Jose, CA, USA).

### 4.6. Intracellular ROS Production

Cells were harvested and suspended in culture media in a 15 mL conical tube. After that, cells were treated with CTL for 1 h and then stained with 20 µM DCFH-DA at 37 °C for 30 min. The fluorescence intensity was measured using flow cytometry (FACSCalibur, BD Biosciences, San Jose, CA, USA) and a microplate reader (Synergy H1, BioTek, Seoul, Republic of Korea).

Mitochondrial ROS levels were detected using the MitoSOX Red reagent (Invitrogen, Carlsbad, CA, USA). Cells were seeded in 6-well plates and treated with CTL for 1 h. Cells were harvested, washed with 1× Hank’s Balanced Salt Solution (HBSS) (ATCC, Sparks, MD, USA), then stained with 1 µM MitoSOX dye for 30 min at 37 °C. The levels of mitochondrial ROS were analyzed by flow cytometry.

### 4.7. Loss of Mitochondrial Membrane Potential (MMP)

After CTL treatment for 24 h, cells were stained with Rho 123 (1 µg/mL) at 37 °C for 1 h. To estimate MMP (ΔΨm), cells were harvested, resuspended in PBS, and analyzed using flow cytometry (FACSCalibur, BD Biosciences, San Jose, CA, USA).

### 4.8. Acridine Orange (AO) Staining

Cells were treated with CTL for 24 h and stained with 2 µg/mL AO at 37 °C for 10 min. For confocal microscopy, cells were fixed with 4% for paraformaldehyde (PFA) for 10 min and washed with PBS. Images were acquired by confocal microscopy (Carl Zeiss, Jena, Germany). For flow cytometry, the stained cells were harvested, resuspended with PBS, and analyzed using flow cytometry (FACSCalibur, BD Biosciences, San Jose, CA, USA). The fluorescence intensity was measured via the FL-1 channel.

### 4.9. Plasmid and Transfection

To generate LC3-overexpressed cells, pQCXI Puro DsRed-LC3-GFP provided by D. Sabatini (Addgene plasmid #31182, Cambridge, MA, USA) was used. Cells were transfected with 2 µg GFP-LC3 using Lipofectamine 3000 (Invitrogen, Carlsbad, CA, USA). For the knockdown of PINK1 and Parkin, cells were transfected with 30 pmol Parkin siRNA (5′-GCCACGUGAUUUGCUUAGATT-3′) using RNAiMAX (Invitrogen, Carlsbad, CA, USA). As a negative control, control siRNA was purchased from Santa Cruz Biotechnology. Transfected cells were harvested, seeded, and used for further assay.

### 4.10. Confocal Microscopy

After CTL treatment for 24 h, breast cancer cells were fixed with 4% PFA in PBS and permeabilized with 0.5% Triton X-100 for 7 min. After PBS washing, cells were blocked with PBS-T containing 10% FBS and 1% BSA for 1 h. To measure the release of cathepsin D into the cytosol, cells were incubated with anti-cathepsin D primary antibody (sc-6486, 1:200, Santa Cruz Biotechnology, Santa Cruz, CA, USA) overnight and followed by Alexa Flour 488-conjugated anti-goat IgG for 1 h (1:250, Invitrogen, Carlsbad, CA, USA). Nuclei were stained with 1 μg/mL DAPI (Sigma, St. Louis, MO, USA) in 2% BSA for 1 min. To analyze the mitophagy induction through the interaction of mitochondria with the lysosome, cells were incubated with primary antibodies (anti-COX Ⅳ, #4850, 1:250, Cell Signaling, Danvers, MA, USA and anti-LAMP2, sc-18822, 1:250, Santa Cruz Biotechnology, Santa Cruz, CA, USA). Cells were incubated with Alexa Flour 488-conjugated anti-rabbit IgG and 594-conjugated anti-mouse IgG (1:250, Invitrogen, Carlsbad, CA, USA) for 1 h. The autophagosome-mediated mitophagy was analyzed by staining with anti-COX Ⅳ in GFP-LC3-transfected cells, followed by incubating with Alexa Flour 594-conjugated anti-rabbit IgG. To assess the integrity of lysosome, cells were treated with CTL for 24 h and stained with 50 nM Lysotracker Red DND-99 at 37 °C for 30 min. Fluorescence images were acquired using confocal microscopy (Carl Zeiss, Jena, Germany).

### 4.11. Immunoprecipitation (IP) Assay

Cells were lysed with IP lysis buffer (Thermo Scientific, Waltham, MA, USA) containing protease and phosphatase inhibitors. At centrifugation, the lysates were precleared with protein A/G agarose bead (Thermo Scientific, Waltham, MA, USA) at 4 °C for 1 h, and supernatants were incubated with anti-Parkin overnight at 4 °C. The supernatants were immunoprecipitated with 20 μL of protein A/G agarose bead at 4 °C for 1 h, and the formed complexes were washed three times with washing buffer (25 mM Tris-HCl, 150 mM NaCl, and 1% NP-40). After removing the last supernatant, the bead was prepped for western blotting by boiling at 100 °C for 10 min in 2× sample buffer.

### 4.12. Statistical Analysis

Data are represented as the mean ± standard deviation (SD). Statistical analysis was tested using the Student’s *t*-test or one-way ANOVA with Bonferroni post-hoc test and used GraphPad Prism 5.0 (GraphPad Software Inc., CA, USA). *p* values < 0.05 were regarded as a significant difference.

## Data Availability

All data and materials are described within the article.

## Supplementary Materials

The following supporting information can be downloaded at: https://www.mdpi.com/article/10.3390/ijms24044009/s1.
