# Supplementary material for: Investigation of Molecular Mechanisms Involved in Sensitivity to the Anti-Cancer Activity of Costunolide in Breast Cancer Cells
Source: Int J Mol Sci. 2023 Feb 16;24(4):4009. doi: 10.3390/ijms24044009 (PMC9965698; doi:10.3390/ijms24044009)
Supplement: Supplementary file 1 [file ijms-24-04009-s001.zip › ijms-1980002-supplementary.pdf]

International Journal of *Molecular Sciences*

*Supplementary figures*

# Investigation of molecular mechanisms involved in susceptibility to the anti-cancer activity of costunolide in breast cancer cells

**Yu-Jeong Choi [1], Youn Kyung Choi [2], Seong-Gyu Ko [3], Chunhoo Cheon [3,*] and Tai Young Kim [4,*]**

1 Department of Science in Korean Medicine, Graduate School, Kyung Hee University, Seoul, 02447, Korea
2 Jeju Research Center for Natural Medicine, Jeju National University, Jeju, 63243, Korea
3 Department of Preventive Medicine, College of Korean Medicine, Kyung Hee University, Seoul, 02447, Korea
4 Center for Cognition and Sociality, Institute for Basic Science, Daejeon, 34126, Korea
* Correspondence: hreedom@khu.ac.kr; Tel.: 82-2-961-0329, taik@ibs.re.kr; Tel: 82-42-878-9155

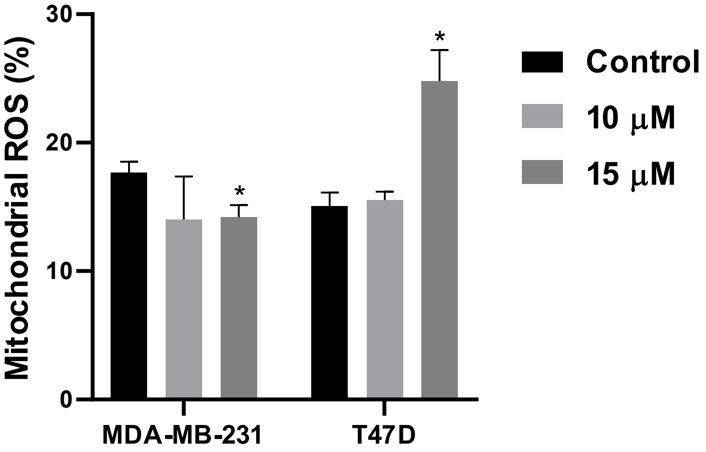


**Figure S1.** Mitochondrial ROS levels in breast cancer cells. Cells were treated with CTL for 1 h and stained with MitoSOX dye for 30 min. ROS levels were detected by flow cytometry (FACSCalibur, BD Biosciences, USA). Data represent the mean ± SD. *, P < 0.05 by Student's t-test.

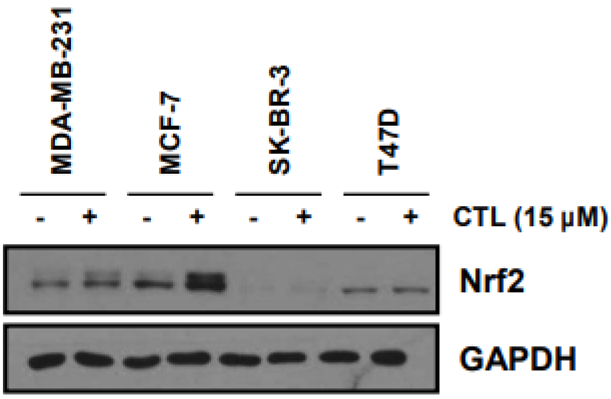


**Figure S2.** The protein levels of Nrf2 in breast cancer cells. Cells were treated with CTL (15 μM) for 1 h. Western blot analysis was performed using an Nrf2 antibody (#12721, Cell Signaling Technology).
